# Lipofection of Non-integrative CRISPR/Cas9 Ribonucleoproteins in Male Germline Stem Cells: A Simple and Effective Knockout Tool for Germline Genome Engineering

**DOI:** 10.3389/fcell.2022.891173

**Published:** 2022-06-14

**Authors:** Mariella Obermeier, Jim Vadolas, Stefaan Verhulst, Ellen Goossens, Yoni Baert

**Affiliations:** ^1^ Biology of the Testis Lab, Vrije Universiteit Brussel (VUB), Brussels, Belgium; ^2^ Department of Molecular and Translational Sciences, Monash University, Clayton, VIC, Australia; ^3^ Centre for Cancer Research, Hudson Institute of Medical Research, Clayton, VIC, Australia; ^4^ Liver Cell Biology Research Group, Vrije Universiteit Brussel (VUB), Brussels, Belgium

**Keywords:** gene editing, CRISPR/Cas, germline stem cells, ribonucleoproteins (RNPs), germline genome editing, gene knockout, gene correction, spermatogonial stem cell (SSC)

## Abstract

Gene editing in male germline stem (GS) cells is a potent tool to study spermatogenesis and to create transgenic mice. Various engineered nucleases already demonstrated the ability to modify the genome of GS cells. However, current systems are limited by technical complexity diminishing application options. To establish an easier method to mediate gene editing, we tested the lipofection of site-specific Cas9:gRNA ribonucleoprotein (RNP) complexes to knockout the enhanced green fluorescent protein (*Egfp*) in mouse EGFP-GS cells via non-homologous end joining. To monitor whether gene conversion through homology-directed repair events occurred, single-stranded oligodeoxynucleotides were co-lipofected to deliver a *Bfp* donor sequence. Results showed *Egfp* knockout in up to 22% of GS cells, which retained their undifferentiated status following transfection, while only less than 0.7% EGFP to BFP conversion was detected in gated GS cells. These data show that CRISPR/Cas9 RNP-based lipofection is a promising system to simply and effectively knock out genes in mouse GS cells. Understanding the genes involved in spermatogenesis could expand therapeutic opportunities for men suffering from infertility.

## Introduction

Gene editing in the male germline is a potent tool to study function and failure of spermatogenesis, to create animal models and to expand therapeutic opportunities for men suffering from infertility (Mulder et al., 2016; Vassena et al., 2016; Wang et al., 2017). Spermatogonial stem cells are the most primitive postnatal germ cells and characterized by self-renewal and differentiation into fertilization-capable spermatozoa (Kubota and Brinster, 2018). Cultured mouse spermatogonial stem cells, aka mouse “germline stem” (GS) cells (Kanatsu-Shinohara et al., 2003), are able to proliferate more than 2 years while maintaining their spermatogenic potential and (epi)genetic stability (Kanatsu-Shinohara et al., 2005). These properties present opportunities and can be leveraged in gene editing experiments that study the basis of genetic infertility. Further, as GS cell clones grow in colonies, they are ideal to control off-target effects and to identify desired genetic modifications after gene editing, which is especially beneficial when developing therapeutic applications or animal models of disease (Vassena et al., 2016; Kubota and Brinster, 2018). Today, genetically modified animal models are often created by manipulating zygotes or embryos. However, to overcome the limitations of mosaicism formation and ethical concerns associated with that, in many cases germline genome engineering in GS cells could represent an excellent alternative (Shao et al., 2014; Vassena et al., 2016; Takashima, 2018).

Specific editing in the genome can be driven by engineered nucleases including zinc-finger nucleases (ZFNs), transcription activator-like effector nucleases (TALENs) and clustered regularly interspaced short palindromic repeats/CRISPR-associated protein (CRISPR/Cas) (Gupta and Musunuru, 2014). The systems target the DNA precisely and produce site-specific double strand breaks, stimulating the main DNA-repair mechanisms, non-homologous end joining (NHEJ) and homology-directed repair (HDR). This machinery can be used to create gene knockouts and mutations (via NHEJ) and specific insertions by incorporating an appropriate DNA template (via HDR) (Gaj et al., 2013). In mouse GS cells, ZFNs showed a very low editing efficiency, whereas TALENs and CRISPR/Cas9 reached higher, and comparable, editing rates (Fanslow et al., 2014; Sato et al., 2015). However, the CRISPR/Cas9 system is generally considered as a faster, cheaper and more feasible approach to modify the genome (Broeders et al., 2020). Several studies demonstrated that CRISPR/Cas9 altered GS cells were able to differentiate *in vivo* and to produce healthy non-mosaic offspring carrying the desired genetic modification (Chapman et al., 2015; Sato et al., 2015; Wu et al., 2015).

The success of gene editing also depends on the delivery routes, guiding transfection complexes efficiently into the targeted cells without inducing cell damage. The CRISPR/Cas9 system can be delivered in the form of DNA, RNA or (ribonucleo-) proteins (Broeders et al., 2020). Most CRISPR/Cas9 studies in rodent GS cells are based on the electroporation of plasmids (Chapman et al., 2015; Sato et al., 2015; Wu et al., 2015; Li et al., 2019). However, plasmids are limited by safety concerns due to integration risk, extended off-target activity and higher cell death compared to its alternatives (Li et al., 2018; Hsu et al., 2019).

In this study, we focused on testing a non-integrative and simple system to mediate efficient gene editing in GS cells, being Cas9:gRNA ribonucleoprotein (RNP) complexes delivered by lipofection. This has been shown to be efficient in cultured human cells (Zuris et al., 2015). The non-integrative protein:RNA RNP complexes act quick and were shown to be efficient in “hard-to-transfect” cells (Kim et al., 2014). In addition, lipid-mediated transfection does not require special equipment and is consequently easier to use and cheaper than alternative viral and non-viral choices.

To rapidly and easily study gene knockout (via NHEJ) and gene correction (via HDR), the enhanced green fluorescent protein (EGFP) to blue fluorescent protein (BFP) conversion method was explored in murine EGFP-GS cells. Following gene editing, NHEJ and HDR of *Egfp* can be simultaneously quantified by the loss of EGFP and gain of BFP fluorescence, respectively (Glaser et al., 2016). Here, EGFP to BFP conversion can be equated to gene correction, as the underlying HDR mechanism is also used to correct defective genes. As the originally EGFP to BFP gene editing method was performed by electroporation of plasmids, we first explored gene editing using lipofection of Cas9:gRNA RNP and DNA templates in EGFP-HEK293T cells. Our results suggest that CRISPR/Cas9 RNP-based lipofection represents a promising system to induce simple and effective gene knockouts in GS cells.

## Materials and Methods

### Animals

Testicular cells were isolated from the F1 generation (B6D2F1/2) of female C57BL/6-Tg (CAG-EGFP)13Osb/LeySopJ and male DBA/2J mice (both purchased from Charles River Laboratories; Brussels, Belgium), as GS cell lines can be efficiently established from these strains (Kanatsu-Shinohara and Shinohara, 2010). In B6D2F1/2 mice, EGFP was expressed in all cells containing a nucleus and controlled by a cytomegalovirus enhancer and a chicken β-actin promotor. Approval for breeding and testis collection was given by the Ethical Committee of the *Vrije Universiteit Brussel* (20-216-2 & 20-216-OC1).

Testes were isolated from prepubertal mice at day 5–7 post-partum. The tunica albuginea was removed and testicular tissue was cryopreserved and thawed as described in Baert et al. (2012).

### Derivation and Maintenance of a GS Cell Culture

An EGFP-GS cell line was derived based on the protocol of Kanatsu-Shinohara et al. (Kanatsu-Shinohara and Shinohara, 2010). Briefly, testes were enzymatically digested using collagenase I (1 mg/ml) and trypsin, filtered through a 70 μm cell strainer and finally seeded into a 0.4% gelatine coated 12-well plate (start concentration 5 × 10^4^ cells/cm^2^ in 0.8 ml GS cell medium). Germ and somatic cells were separated via differential plating by only transferring floating germ cells and weakly adhered somatic cells to a non-coated 12-well plate after overnight incubation. Cells were passaged 1:1 (P1) and 1:2 (P2) every 10–14 days, resulting in an almost purified GS cell population. After the second passage, cells were placed onto mitomycin-inactivated mouse embryonic fibroblasts (MEFs; A34962; Thermo Fisher Scientific; Merelbeke, Belgium), cultured at a density of 5 × 10^4^ cells/cm^2^ (37°C, 5% CO_2_) and passaged when GS cell culture reached confluency. Medium was prepared as described in Kanatsu-Shinohara et al. (Kanatsu-Shinohara and Shinohara, 2010) and changed every 2–3 days. Cell culture was followed up using an inverted fluorescence microscope (Olympus; Berchem, Belgium) and the software ToupView (ToupTek Photonics; Hangzhou, China).

### Cas9:gRNA Complexing

Cas9 and gRNA were purchased from Integrated DNA Technologies (IDT; Leuven, Belgium) as Alt-R^®^ S.p. Cas9 Nuclease V3 (224675276) and Alt-R^®^ CRISPR-Cas9 sgRNA. CRISPR gRNA (5′-CTC​GTG​ACC​ACC​CTG​ACC​TA-3′) targeting *Egfp* was prior designed and evaluated by Glaser et al. (Glaser et al., 2016). Cas9 and gRNA were dissolved in Opti-MEM and nuclease free IDTE buffer (pH7), respectively, and complexed by incubating the dissolved compounds in Opti-MEM (5min, room temperature [RT]).

### Single-Stranded Oligodeoxynucleotides

Notably, the conversion of *Egfp* to *Bfp* requires only one base pair replacement (196T > C substitution) (Glaser et al., 2016). HDR of *Egfp* in the presence of a specifically modified donor DNA template (5′-ACC​CTG​AAG​TTC​ATC​TGC​ACC​ACC​GGC​AAG​CTG​CCC​GTG​CCC​TGG​CCC​ACC​CTC​GTG​ACC​ACC​CTG​AGC​CAC​GGG​GTG​CAG​TGC​TTC​AGC​CGC​TAC​CCC​GAC​CAC​ATG​AAG​CAG​CAC​GAC​TTC​TTC​AAG​TCC​GCC​ATG​CC-3′) resulted in a fluorescence absorption and emission shift toward the blue spectrum, thus, creating BFP (Glaser et al., 2016). The repair template was delivered in the form of single-stranded oligodeoxynucleotides (ssODN) (Ultramer^®^ DNA Oligo; IDT). The same sequence as “ssODN2” in Glaser et al. was used (Glaser et al., 2016).

### RNAiMAX-Mediated Transfection

Cells were transfected following the manufacturer´s recommendation for Cas9:gRNA RNP transfection (IDT). RNP complexes and ssODN were mixed in Opti-MEM with RNAiMAX (2.5 μL/cm^2^; 13778030; Thermo Fisher Scientific) and incubated for 20 min (RT).

Before proceeding with GS cells, we first performed a proof-of-concept in EGFP-HEK293T cells, which were stably transduced with an integration competent lentiviral EGFP expression construct as done before by Glaser et al. (Glaser et al., 2016). The cells (8 × 10^4^/cm^2^) were reverse transfected with 10 nM RNP and 42 nM ssODN. Culture medium consisted of DMEM supplemented with L-glutamine (6 nM), 10% FBS and 1% Pen/Strep. Cells were transfected in medium without antibiotics. Transfection complexes were removed after 6 h incubation and medium was changed to medium with antibiotics.

EGFP-GS cells (8.42 × 10^4^ cells/cm^2^, P6) were incubated with differently concentrated transfection complexes including either 3, 10 or 30 nM RNP and 42, 84 or 126 nM ssODN. The cells were reverse transfected in GS cell medium (105 μL/cm^2^) without antibiotics in feeder-free condition for 6 h to avoid interference from MEFs. Medium with cells and transfection complexes was then transferred onto a MEF feeder. Transfection was stopped after 24 h by replacing the transfection medium with fresh GS cell medium without antibiotics. Medium was changed to GS cell medium with antibiotics 48 h after transfection onset.

Each condition was tested with triplicates. Two controls were included, comprising the negative control (UNTR = untreated) and an unloaded RNAiMAX control (MOCK). Gene knockout was targeted by RNP-loaded RNAiMAX (RNP) and gene conversion by RNP/ssODN-loaded RNAiMAX (RNP + ssODN). Gene editing was visually followed up by using an inverted fluorescence microscope and the software ToupView.

### Flow Cytometry

Gene knockout (loss of EGFP expression) and gene conversion (BFP expression) in HEK293T and GS cells were qualitatively assessed by measuring EGFP/BFP fluorescence. Cells were trypsinized and collected in 5 ml phosphate buffered saline (PBS). Clumps and debris were excluded from the analysis based on scatter characteristics. Dead cells were identified and removed based on 7-aminoactinomycin D positivity (420403; Biolegend; Amsterdam, Netherlands). GS cells were furthermore gated based on their specific light-scattering characteristics in the side scatter versus forward scatter dot plots (Kanatsu-Shinohara et al., 2013). Thus, only the gated non-debris, singlets, living HEK/GS cell-phenotype was included into the EGFP/BFP dot plot analysis.

GS cell fraction was calculated by dividing the number of cells presenting the GS cell phenotype by all detected cells under the flow cytometer (GS cell phenotype/total cells present) and expressed in percentage (x 100%).

### Histology and Immunofluorescence Staining

To study whether the stem cell phenotype was influenced by the gene editing procedure, GS cell cultures were evaluated for their expression of the general germ cell marker DEAD-box helicase 4 (DDX4) and the early germ cell marker undifferentiated embryonic cell transcription factor 1 (UTF1) (Castrillon et al., 2000; van Bragt et al., 2008). Ten days after transfection, cells were fixed in PBS-4% formaldehyde (10 min) and washed in PBS (3 × 5 min). Cell membranes were permeabilized by incubating in 0.1% Triton X-100 and 0.1% sodium citrate in PBS (20 min, 4°C). Unspecific binding sites were blocked with 10% normal donkey serum (1 h, RT). The primary antibodies rabbit anti-DDX4 (2.5 μg/ml; ab13840; Thermo Fisher Scientific) and mouse anti-UTF1 (20 μg/ml; MAB4337; Sigma; Overijse, Belgium) were diluted in PBS-1% bovine serum albumin and incubated overnight. At the next day, the cells were washed in PBS (3 × 5 min) and incubated with donkey anti-rabbit Alexa Fluor 647 (10 μg/ml; A-31573; Thermo Fisher Scientific) and donkey anti-mouse Alexa Fluor 555 (10 μg/ml; A-31570; Thermo Fisher Scientific) secondary antibodies (1 h, RT). Afterwards, the cells were washed in PBS (3 × 5 min), coated with ProLong™ Gold Antifade Mountant (P36934; Thermo Fisher Scientific) and sealed with cover glass. Pictures were captured using an inverted fluorescence microscope and edited with the software Cell^F (Olympus).

### Statistical Analysis

Quantitative results are presented as means ± standard deviation. Statistical analysis and graphics were created using the software GraphPad Prism 9.3.1. A *p*-value < 0.05 was considered statistically significant. Normality was assessed using the D´Agostino-Pearson omnibus (K2) test, and homogeneity of variances using the Brown-Forsythe test. Statistical significance for gene knockout in HEK293T cells as well as gene knockout and gene conversion in GS cells was evaluated by one-way ANOVA followed by a multiple comparison Turkey´s test. A Kruskal-Wallis test followed by a multiple comparison Dunn´s test was applied for gene conversion in HEK293T cells.

## Results

### HEK293T Cells: Gene Knockout and Gene Conversion

**FIGURE 1:**
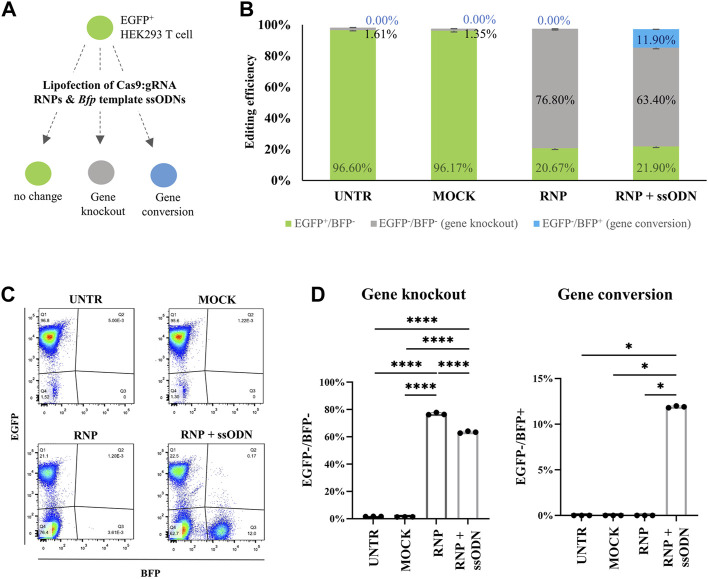
Evaluation of gene editing in HEK293T cells by flow cytometry. **(A)** Cas9:gRNA RNP (10 nM) and *Bfp* template ssODN (42 nM) were lipofected in EGFP-HEK293T cells to test gene knockout (EGFP^−^/BFP^−^) and gene conversion (EGFP^−^/BFP^+^). **(B)** Gene knockout (EGFP^−^/BFP^−^, grey bars), gene conversion (EGFP^−^/BFP^+^, blue bars) and unaffected cells (EGFP^+^/BFP^−^, green bars) in the HEK293T cell experiment. **(C)** Representative EGFP/BFP dot plots 10 days after transfection. Lipofecting RNP alone stimulated a solid gene knockout (EGFP^−^/BFP^−^) population (RNP). Co-lipofecting RNP and ssODN (RNP + ssODN) induced both, gene knockout and gene conversion (EGFP^−^/BFP^+^). The UNTR and MOCK control remained unaffected (EGFP^+^/BFP^−^). **(D)** Gene knockout (EGFP^−^/BFP^−^) percentage in RNP was significantly higher compared with RNP + ssODN, UNTR and MOCK. Statistical significance was determined using a one-way ANOVA, followed by a multiple comparison Turkey´s test. Gene conversion (EGFP^−^/BFP^+^) was detected after RNP and ssODN (RNP + ssODN) co-lipofection and completely absent in the UNTR, MOCK and RNP controls. Statistical evaluation was performed using a Kruskal-Wallis test followed by a multiple comparison Dunn´s test. UNTR: negative control; MOCK: unloaded RNAiMAX; RNP: RNAiMAX loaded with RNP; RNP + ssODN: RNAiMAX loaded with RNP and ssODN. *p < 0.05; ****p < 0.0001. n = 3 technical replicates.

**FIGURE 2:**
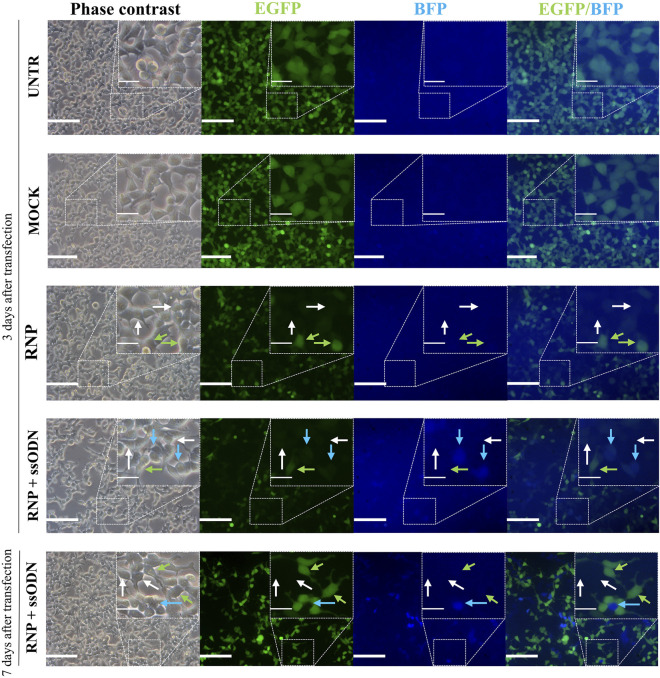
Visual evaluation of gene editing in HEK293T cells. Three days after transfection, gene knockout (EGFP^−^/BFP^−^) and gene conversion (EGFP^−^/BFP^+^) were observed under a fluorescence microscope. This was even better detectable 7 days after transfection. Gene knockout (EGFP^−^/BFP^−^, grey arrows) was detected in RNP and RNP + ssODN, but absent in the MOCK and UNTR control. Gene conversion (EGFP^−^/BFP^+^, blue arrows) was only observed after co-lipofecting ssODNs (RNP + ssODN). Unaffected cells (EGFP^+^/BFP^−^, green arrows) were detected in all conditions. UNTR: negative control; MOCK: unloaded RNAiMAX; RNP: RNAiMAX loaded with RNP; RNP + ssODN: RNAiMAX loaded with RNP and ssODN. Scale bars represent 100 µm in whole pictures and 25 µm in inserts. Inserts depict higher magnifications of the boxed area in the pictures.

Cas9:gRNA RNP (10 nM), directed against *Egfp*, and ssODN (42 nM), a donor template for *Bfp*, were lipofected in EGFP-HEK293T cells. Ten days after transfection, EGFP and BFP expression was assessed by flow cytometry to quantify NHEJ and HDR, corresponding to gene knockout (EGFP^−^/BFP^−^) and gene conversion (EGFP^−^/BFP^+^), respectively (Figures 1A–C). Data showed 76.80 ± 0.87% non-fluorescent cells (EGFP^−^/BFP^−^) in the RNP condition, which is significantly higher than 1.61 ± 0.09% in UNTR (*p* < 0.0001), 1.35 ± 0.05% in MOCK (*p* < 0.0001) and 63.40 ± 0.70% in RNP + ssODN (*p* < 0.0001), indicating efficient *Egfp* knockout (Figure 1D). EGFP to BFP conversion, associated with HDR, was detected in 11.90 ± 0.10% HEK293T cells after co-lipofecting RNP and ssODN (Figure 1D). In contrast, no BFP-expressing cells were detected in the UNTR (0.00 ± 0.00%, *p* < 0.05), MOCK (0.00 ± 0.00%, *p* < 0.05) and RNP (0.00 ± 0.00%, *p* < 0.05) control conditions (Figure 1D). Loss of EGFP expression and EGFP to BFP conversion was confirmed by fluorescence microscopy (Figure 2).

### GS Cells: Culture Efficiency After Transfection

**FIGURE 3:**
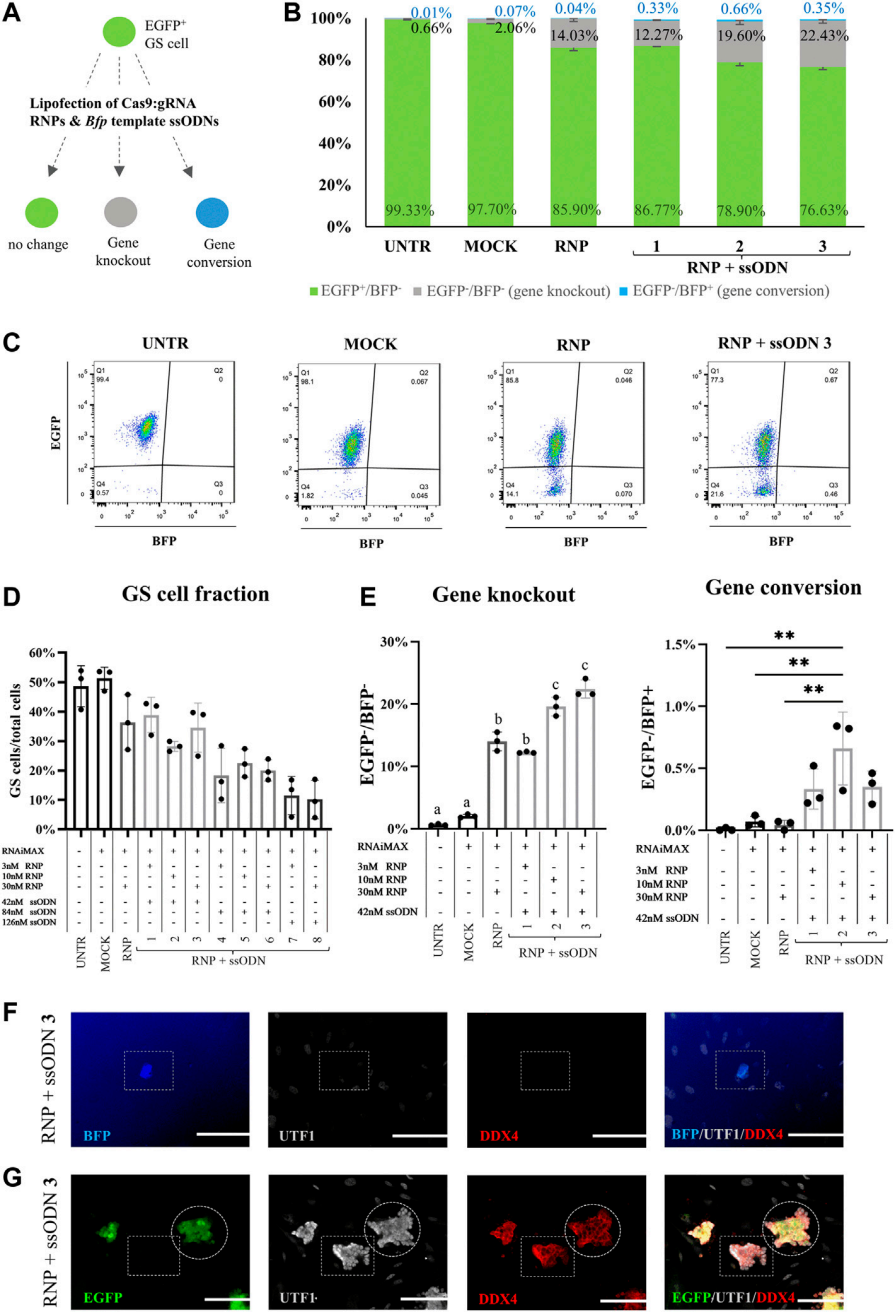
Gene editing in GS cells. **(A)** Cas9:gRNA RNP and *Bfp* template ssODN were lipofected in GS cells to test gene knockout (EGFP^−^/BFP^−^) and gene conversion (EGFP^−^/BFP^+^). **(B)** Gene knockout (EGFP^−^/BFP^−^, grey bars), gene conversion (EGFP^−^/BFP^+^, blue bars) and unaffected cells (EGFP^+^/BFP^−^, green bars) in the GS cell gate, 10 days after transfection. **(C)** Representative EGFP/BFP dot plots 10 days after transfection. Flow cytometry detected gene knockout (EGFP^−^/BFP^−^) in conditions lipofected with RNP and RNP + ssODN but not in the UNTR and MOCK control. Co-lipofection of RNP and ssODN caused only gene conversion in a small fraction of the gated GS cell phenotype. **(D)** Calculating the GS cell fraction (GS cells/all cells present in culture) revealed that high concentrations of ssODN reduced the GS cell fraction, while RNAiMAX treatment (MOCK) had no effect and RNP administration (RNP) only a slightly reducing effect on the GS cell fraction. Due to the low GS cell fraction (>2-fold decrease), conditions 4–8 were excluded from further analysis. **(E)** Gene knockout (EGFP^−^/BFP^−^) was detected in all conditions which were transfected with RNP (RNP and RNP + ssODN 1–3). Condition 3 was identified as the best condition, with a significant increase in *Egfp* knockout compared to UNTR, MOCK, RNP and RNP + ssODN 1. Statistical significance was assessed performing a one-way ANOVA followed by a multiple comparison Turkey´s test. ^a,b,c^p≥0.05. RNP vs. 2: *p* < 0.001. All other comparisons: *p* < 0.0001. Gene conversion was detected after co-lipofecting RNP and ssODN, but only in <0.70% of the population in the GS cell gate. Statistical significance was tested using a one-way ANOVA, followed by a multiple comparison Turkey´s test. **p < 0.01. **(F)** BFP-expressing cells were detected in the culture, but ultimately not identified as GS cells, as they did not express their specific marker (UTF^−^/DDX4^−^). **(G)** EGFP^+^ and EGFP^−^ GS cell colonies maintained the undifferentiated germ cell status (UTF1^+^/DDX4^+^) 10 days after transfection. n = 3 technical replicates. Scale bars represent 100 µm.

After confirming robust gene knockout and conversion in HEK293T cells, the system was applied in GS cells (Figure 3A). Different concentrations of RNP (3, 10 and 30 nM) and ssODN (42, 84 and 126 nM) were tested to determine the effect of the dose and ratio on gene editing. Ten days after transfection, culture efficiency was assessed by determining the GS cell fraction (GS cell phenotype/all cells present in culture) based on flow cytometry gates. Compared with UNTR (48.60 ± 6.97%), numerically, flow cytometry revealed no decline of the GS cell fraction in MOCK (51.33 ± 3.75%), a slight decrease in RNP (36.37 ± 9.35%) and RNP + ssODN conditions 1–3 (28.20-38.87 ± 1.70-8.35%). Increased ssODN concentrations resulted in a considerable loss (>2-fold) of the GS cell fraction, down to 10.17 ± 6.53% in the most extreme condition (Figure 3D). Consequently, the latter conditions were excluded from further analysis (RNP + ssODN, condition 4–8). This was confirmed by fluorescence microscopy showing poor to absent GS cell colony growth in condition 4–8 (RNP + ssODN, Supplementary Figure S1).

### GS Cells: Gene Knockout and Gene Conversion, Cell Functionality

At culture day eleven, *Egfp* knockout was detected in 14.03 ± 1.50% of the GS cells when lipofecting RNP alone (Figures 3B,C). This was significantly higher compared to 0.66 ± 0.14% in UNTR (*p* < 0.0001) and 2.06 ± 0.28% in MOCK (*p* < 0.0001, Figure 3E). Co-lipofection with ssODN resulted in a significantly increased *Egfp* knockout, up to 22.43 ± 1.44% in the most ideal condition (condition 3: 30 nM RNP +42 nM ssODN), compared to RNP (*p* < 0.0001), UNTR (*p* < 0.0001), MOCK (*p* < 0.0001) and RNP + ssODN1 (12.27 ± 0.12%, *p* < 0.001, Figure 3E).

Interestingly, only <0.70% BFP-expressing cells were detected in the GS cell fractions (Figures 3B,C), which were also observed under the fluorescence microscope. These cells were presumable residual testicular somatic cells as they did not show the typical GS cell phenotype and did neither express the early germ cell marker UTF1 nor the general germ cell marker DDX4 (BFP^+^/UTF1^−^/DDX4^−^, Figure 3F).

Since stressful culture conditions can trigger loss of spermatogenic potential in GS cells (Kanatsu-Shinohara et al., 2004), also GS cell colonies were tested on their expression of the germ cell markers UTF1 and DDX4. Immunofluorescence confirmed that both, gene knockout (EGFP^−^) and unchanged EGFP^+^ GS cell colonies maintained their undifferentiated germ cell stage (UTF1^+^/DDX4^+^) 10 days after transfection (Figure 3G).

## Discussion

This study aimed to assess a non-integrative and easy gene editing system in rodent GS cells. A technically simple system involving the lipofection of *Egfp*-specific RNP was tested in EGFP-GS cells for its gene knockout ability. Gene conversion was evaluated in this study by additionally transfecting an ssODN-*Bfp* repair template to induce EGFP to BFP conversion.

To our knowledge, this is the first study investigating Cas9:gRNA RNP lipofection in GS cells. Usually, lipid-mediated delivery is considered as inefficient to transfect stem cells (Zhang et al., 2017). Indeed, Fanslow et al. described significantly lower transfection efficiencies in mouse GS cells after the lipofection of plasmids, compared to electroporation (Fanslow et al., 2014). On the downside, electroporation is typically accompanied by substantial cell death from high voltage pulses of the electroporator, requiring the use of greater quantities of cells compared to chemical transfection methods (Zuris et al., 2015; Hsu et al., 2019). Also, studies using electroporation of TALEN or CRISPR/Cas9 plasmids reported gene knockout percentages up to 18% in murine and porcine spermatogonial stem cells (Chapman et al., 2015; Tang et al., 2018; Li et al., 2019). In contrast, our RNP/ssODN lipofection system induced slightly higher *Egfp* knockout in up to 22% of the mouse GS cells, which is comparable to the results of a recent study of Webster et al., where Cas9:gRNA RNP were electroporated in porcine spermatogonia and caused gene-dependent knockout of 20–35% (Webster et al., 2021). Consequently, our lipofection-mediated system could be a valuable alternative that, additionally, saves costs and technical complexity. On top of that, RNP have the major advantage of being non-integrative and producing less off-target effects (DeWitt et al., 2017), which is important for potential clinical applications.

Loss of EGFP-expression was RNP dose-dependent and did not require the involvement of donor template ssODN. The lipofection of Cas9:gRNA RNP alone (RNP condition) resulted in effective *Egfp* knockout. Co-lipofecting ssODN induced even higher *Egfp* knockout, indicating that ssODN entered the cell nucleus but served as knockout booster instead of acting as *Bfp* template. An explanation for this might be ssODN-mediated cellular responses supporting the NHEJ pathway, as was seen in the co-transfection of non-homologous ssODN (Richardson et al., 2016). Moderate and high concentrations of ssODN caused poor GS cell survival. From this, it seems crucial that, for every new gene editing experiment, the donor template concentration is optimized, to reduce its toxicity. Importantly, the gene editing procedure did not affect the undifferentiated germ cell phenotype, indicating that GS cells maintained their spermatogonial potential. Future studies in which functional genes are targeted should also assess genome integrity (specific and off-target edits), GS cell differentiation and sperm health after the gene editing process to ensure the safety of the offspring.

Intriguingly, co-lipofection of RNP and ssODN did not induce gene conversion in GS cells to the same level as in HEK293T cells, where the *Egfp* knockout and *Egfp* to *Bfp* conversion amounted to 76.80 and 11.90%, in HEK293T cells, respectively. The shift from green to blue fluorescence in the germ cell culture was observed in some residual testicular somatic cells contaminating the culture. However, the balance between NHEJ and HDR events varies amongst species, cell types and cell cycle stages (Shrivastav et al., 2008). Our data suggest that HDR was likely ignored in the mouse GS cells, since no EGFP to BFP conversion was observed in these cells. In fact, extreme rare HDR events have been related to this cell type after inducing DNA double strand breaks (Le et al., 2018), which could explain the differences between HEK293T and GS cell editing in the present study. If required, HDR could be supported by temporary inhibition of molecules which are crucial for NHEJ (Maruyama et al., 2015), optimizations of the ssODN donor template (Liu et al., 2019) or timed delivery of the transfection complexes during the S and G2 phase in which HDR occurs (Lin et al., 2014). Alternatively, prime-editing could be tested to target gene correction in GS cells, since this approach works independent of double strand breaks and its repair pathways (Kantor et al., 2020).

In summary, we report an effective tool for gene knockout in GS cells that could be favorable for large loss-of-function studies, *in vitro* or through the generation of transgenic animals, and the transfection of low cell amounts. The latter is especially of interest for the translation to other species such as the human, in which limited accessibility to GS cells and the lack of reproducible protocols for cell expansion currently hamper their usage for large scale experiments (Baert et al., 2015). Thereby, male germline genome knockouts could have substantial impact to further understand the genetic background of male infertility disorders and to identify possible drug targets.

## Data Availability

The raw data supporting the conclusion of this article will be made available by the authors, without undue reservation.
