# Supplementary material for: Lipofection of Non-integrative CRISPR/Cas9 Ribonucleoproteins in Male Germline Stem Cells: A Simple and Effective Knockout Tool for Germline Genome Engineering
Source: Front Cell Dev Biol. 2022 Jun 14;10:891173. doi: 10.3389/fcell.2022.891173 (PMC9237505; doi:10.3389/fcell.2022.891173)
Supplement: Supplementary file 1 [file DataSheet1.DOCX]

Supplementary Material

#
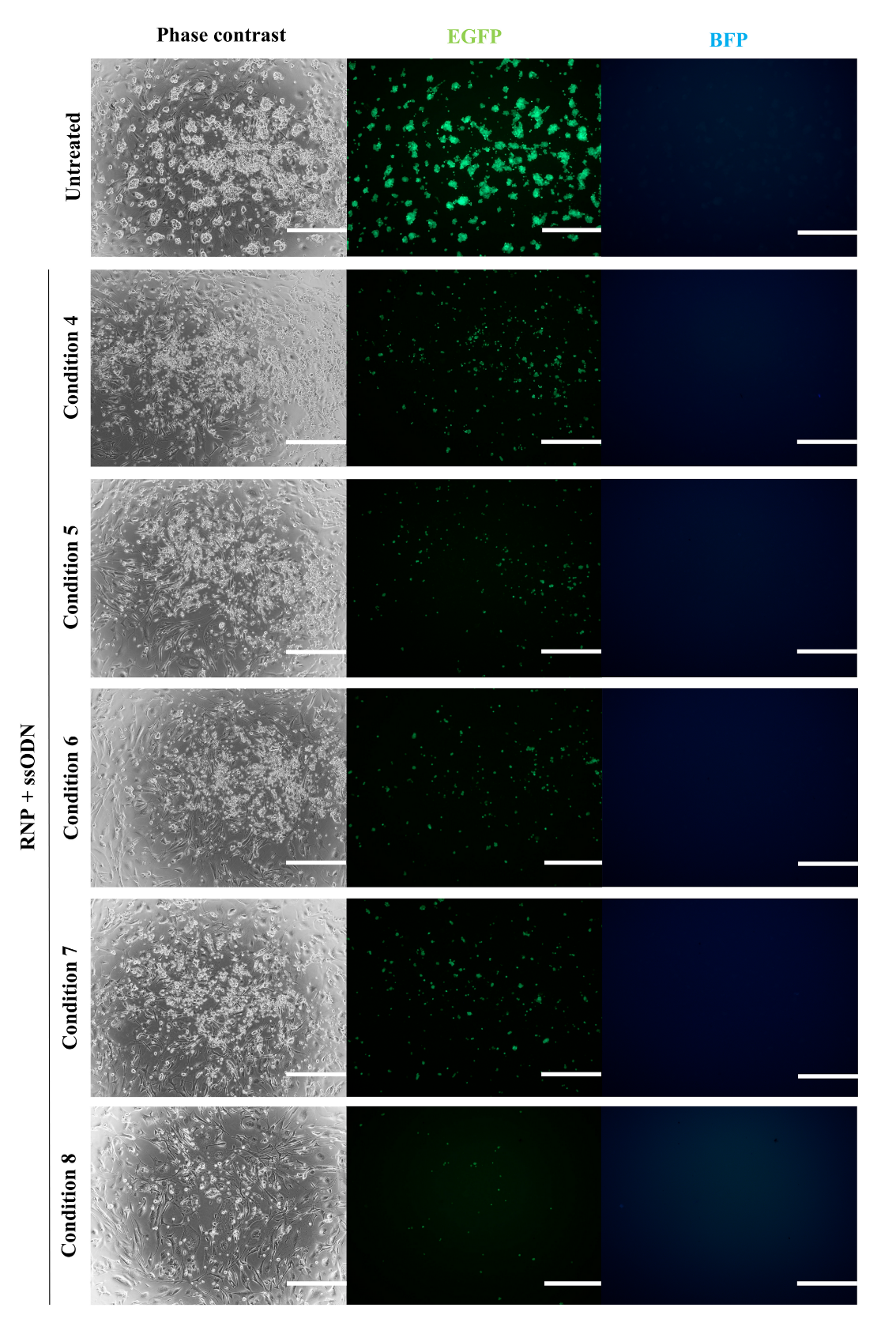
Supplementary Figures

Supplementary Figure 1: Excluded GS cell colonies four days after transfection. Colony growth on MEF feeder cells was clearly impaired in condition 4-8 compared to the UNTR condition. Interestingly, all conditions treated with moderate and high concentrations of ssODN, corresponding to 84nM (condition 4-6) and 126nM (condition 7 & 8), respectively, showed poor GS cell survival. Pictures were taken with a fluorescence microscope. Scale bars represent 400µm.
